# Different Treatments of Symptomatic Angiomyolipomas of the Kidney: Two Case Reports

**DOI:** 10.15586/jkcvhl.v8i4.181

**Published:** 2021-10-19

**Authors:** Giuseppina Pacella, Eliodoro Faiella, Carlo Altomare, Flavio Andresciani, Gennaro Castiello, Caterina Bernetti, Marina Sarli, Bruno Beomonte Zobel, Rosario Francesco Grasso

**Affiliations:** Departmental Faculty of Medicine and Surgery, Università Campus Bio-Medico di Roma, Rome, Italy

**Keywords:** angiomyolipomas, cryoablation, transarterial embolization

## Abstract

Development of more sensitive imaging techniques has caused an increase in the number of diagnosed small renal tumors. Approximately 2–3% of these lesions are proved to be angiomyolipomas (AML), a rare benign tumor of the kidney sometimes causing pain and hematuria. The most required approach is observation, but in the case of recurrent symptoms or larger tumors, which may cause bleeding, a more active treatment is required. We present two cases of symptomatic AML tumors of different sizes in the kidney: one treated with transarterial embolization (TAE), and the other with percutaneous cryoablation (CRA). The lesions were diagnosed on the basis of contrast-enhanced computed tomography (CT) scan and magnetic resonance imaging (MRI). Both treatments proved to be effective and safe for treating renal AMLs. A follow-up carried out, based on contrast-enhanced CT scan, confirmed complete treatment of AML and decreased lesion size. There are myriad minimally invasive approaches for the treatment of renal AMLs, and the preservation of renal function remains a priority. The most popular treatment option is the selective renal artery embolization. Owing to its limited invasiveness, CRA could be an attractive option for the preventive treatment of AML.

## Introduction

Renal angiomyolipoma (AML) is a benign mesenchymal tumor in its classic variant and comprises fat, smooth muscle, and blood vessels. AML accounts for 0.3–3% of all renal masses (1, 2). AML belongs to the group of perivascular epithelioid cell tumors (3). Almost 80% AMLs are sporadic; remaining 20% are associated with the tuberous sclerosis syndrome (TSS) (4).

Historically, 64% of AML were presented symptomatically, most commonly with progressive flank pain, palpable mass, or rupture (5). Contemporary series now indicate that now AML are mostly asymptomatic and incidental lesions, and the increased use of cross-sectional imaging has led to rise in the diagnosis of these lesions (6).

The diagnosis of AML approaches 100% sensitivity with computed tomography (CT) scan or ultrasound. The presence of fat within the lesion indicated on CT scan by a negative attenuation of –10 Hounsfield units or lower, and the sonographic appearance of a hyperechoic signal and acoustic shadowing, is pathognomonic of AML neoplasm (7). Absence of fat in a lesion does not necessarily exclude AML, as it could indicate a lipid-poor AML that can mimic renal cell carcinoma (RCC) (8). Biopsy is rarely required. The primary complication of AML is spontaneous bleeding in the retroperitoneum or into the collecting system (9). The risk of bleeding is related to the angiogenic component of tumor that includes irregular blood vessels (9), and the risk factors of bleeding are related to increase in tumor size (4). There are several options for management of AML: (1) active surveillance (AS); (2) selective arterial embolization (SAE) that can be used to devascularize AMLs (10, 11); (3) surgical removal, preferably by nephron-sparing surgery (NSS) (12); and (4) percutaneous ablation procedures, which is offered in selective patients (9, 13).

The systematic review undertaken by the European Association of Urology (EAU) Renal Cell Carcinoma Guidelines Panel found that AMLs are mostly managed by active surveillance as most of them do not grow (in 89% of cases) (14). Even if they grow, their size increases very slowly and bleeding is a rare event (2.2%). Nevertheless, the 4-cm cut-off, traditionally used as an indication for active treatment, could be reconsidered along with other factors such as patient’s age, rate of growth, and preference of treatment. The most frequently reported active treatment is surgery (31%), particularly NSS, followed by SAE (17%) (14).

## Embolization

Transarterial embolization (TAE) is a first line of treatment for active hemorrhage and symptomatic lesions. TAE is associated with a mean 38.3% (–3.4 cm) reduction in AML size (10). The aim of embolization of AML is twofold: proximal (upstream) embolization to reduce arterial inflow, and distal (tumor bed) embolization (15). A variety of embolic agents have been employed (foam, coil, or microparticle). However, retreatment proportion at 3 years is relatively high at 38% in a pooled analysis of multiple studies (16).

## Ablation

Ablation (percutaneous or laparoscopic) has been performed using a variety of techniques, such as radiofrequency ablation (RFA), microwave ablation, and cryoablation (CRA), for tumors less than 6 cm in size (17).

Castle et al. (18) report a complication rate of 13.3%, and both cryoablation and RFA have a low reintervention rate (0% at 3 years) (16). Although RFA appears to be safe in small- and medium-size renal AMLs, long-term efficacy data are lacking (8).

Microwave ablation is a relatively novel type of thermal ablative procedure that has emerged in recent years; however, currently insufficient data exist on this modality for recommending its routine use for renal AML (8).

For the treatment of renal AMLs, cryoablation has been reported in two studies only with limited number of patients and short-term follow-up (19, 20). These studies indicate substantial progress in the use of cryoablation for the management of small renal masses (SRMs). Makki et al. have demonstrated that cryoablation appears to have a favorable complication profile. With absence of retreatment and a good preservation of renal function, it appears to be a safe and efficient long-term minimal invasive treatment for patients with subclinical renal AMLs to minimize the risk of potentially life-threatening hemorrhage (21).

This report presents two cases of symptomatic renal AML treated with two different techniques.

## Case Report 1

**Figure 1:**
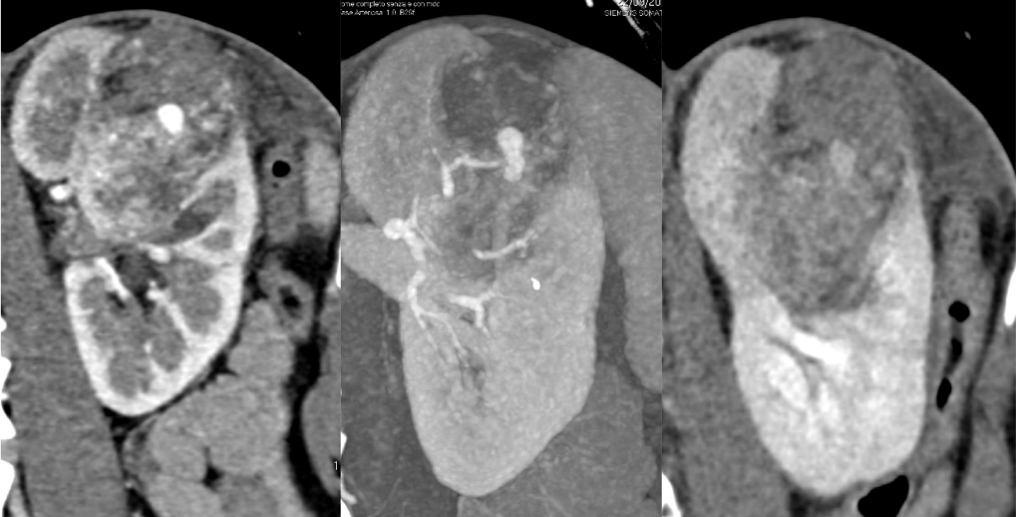
Contrast-enhanced CT scan in coronal reconstruction revealed a 4.5 × 3.5 × 7.0-cm renal AML located in the left kidney, with signs of recent blood component and cellular resentment.

**Figure 2:**
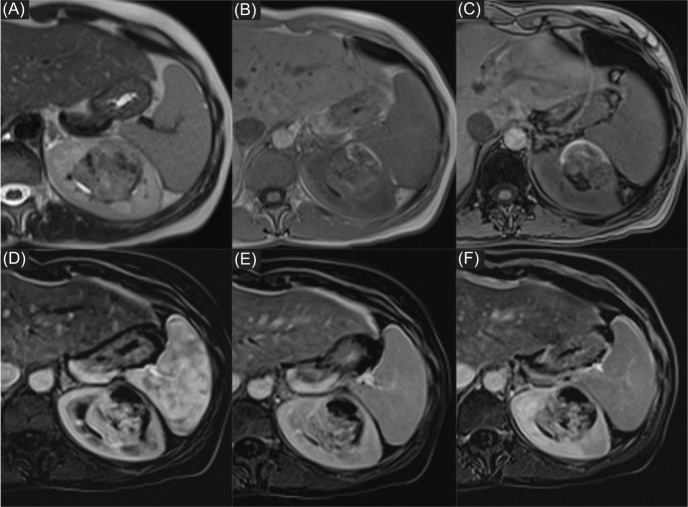
Abdominal MRI demonstrated the presence of an expansive capsulated heterogeneous mass, with inhomogeneous low-to-high signal intensity on (A) T2-weighted images; (B) partial high-signal intensity on in-phase image; and (C) on opposed phase image due to blood content partly and foci of internal fat. (D–F) Subtracted images confirmed an enhancing component in mid-internal portion of mass.

**Figure 3:**
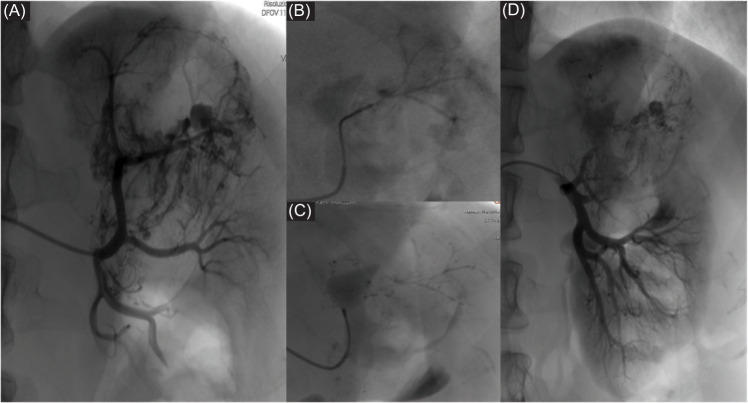
(A) Digital subtraction angiography (DSA) showing a multi-lobulated aneurysm arising from one of the segmental branches of the left renal artery, which was the source of bleeding for this AML. (B–D) DSA obtained post-embolization with coil demonstrating occlusion of the aneurysm.

In September 2017, a 44-year-old female was admitted to our hospital with 1-week history of local left flank pain and macroscopic hematuria. Her laboratory findings such as complete blood cell count and creatinine demonstrated no significant changes. The serum neuron-specific enolase was slightly increased as 24.59 ng/mL (normal range: 0–20 ng/mL). Other tumor markers (serum carbohydrate antigen 125 [CA 125], CA 199, CA 153, CA 724, cytokeratin 19 [CK 19], carcinoembryonic antigen [CEA], and alpha-fetoprotein [AFP]) were within normal range. Contrast-enhanced CT scan revealed a 4.5×3.5×7.0-cm oval mass with heterogeneous density in based scan (CT value: 17–40 HU), located in the left kidney (Figure 1). It contained multiple foci with frank adipose density and a solid component with early and intense enhancement, referable to a renal AML with signs of recent blood component and cellular resentment. Abdominal MRI demonstrated the presence of an expansive capsulated heterogeneous mass, with extension in the context of renal parenchymal, and partial exophytic development in the context of spleno-renal space, with high signal intensity on non-contrast T1-weighted images. It further demonstrated inhomogeneous low-to-high signal intensity on T2-weighted images due to blood content partly and foci of internal fat. In the excretory phase, presence of compressive effects was confirmed, with shift of both upper/middle calyceal groups and renal pelvis (Figure 2). The patient was referred to the interventional radiology service for a selective angiogram and embolization. After selective catheterization of the upper polar arteries of the left kidney using a 5 French catheter, a branch was considered a potential feeder of AML; hence, selective embolization was performed without complications. We used a liquid embolic agent, N-butyl cyanoacrylate-methacryloxy sulfolane (NBCA-MS; Glubran 2^®^; GEM Srl, Viareggio, Italy) with Lipiodol^®^ (Guerbet, Aulnay-Sous-Bois, France) to make the glue radiopaque and modulate the delay of polymerization (Figure 3).

**Figure 4:**
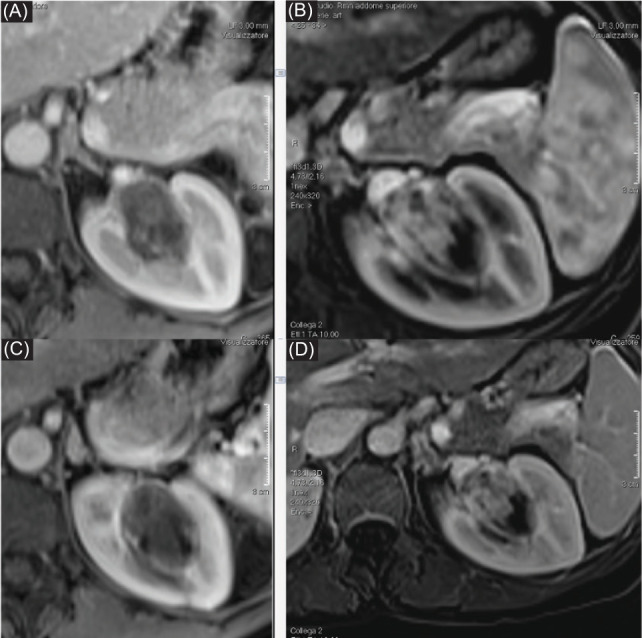
Two-year MRI control: (A, C) T1-weighted contrast-enhanced images, and (B, D) subtracted images demonstrated a reduced size of AML with only a small residual portion vascularized and without damage to healthy renal parenchyma.

No significant change in creatinine levels appeared before and after the procedure. In the follow-up at 6 months of procedure, no bleeding area was observed and the AML was reduced. The patient remained without complaints 2 years after the procedure. The size of the left renal AML was indicated by MRI to be reduced (Figure 4), with only a small residual portion vascularized and without damage to the healthy renal parenchyma.

## Case Report 2

**Figure 5:**
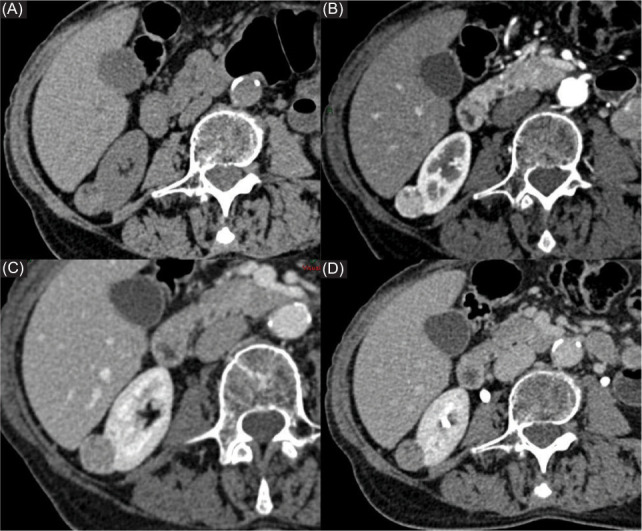
Contrast-enhanced CT scan revealed a 3.0×2.7-cm oval mass in the upper pole of the right kidney with (A) heterogeneous density in based scan due to (B–D) sporadic fat foci and a progressive enhancement observed in the major part of the tumor.

**Figure 6:**
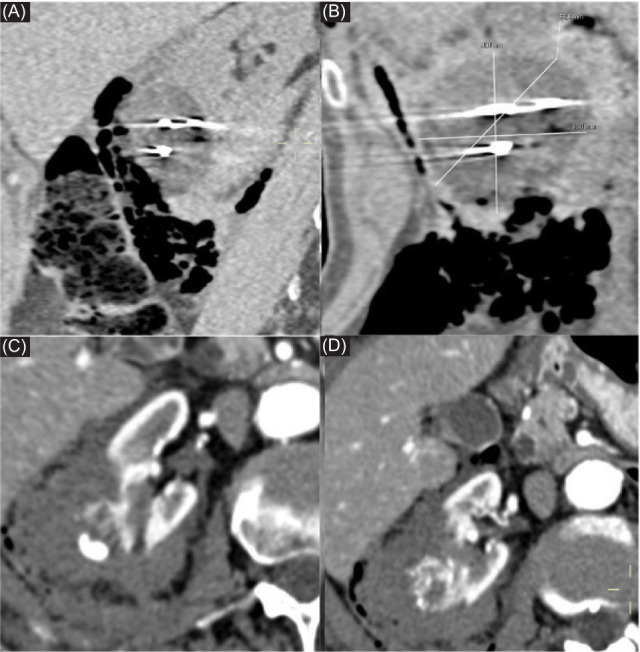
CT scans in (A) axial and (B) coronal reconstruction during the procedure established the intralesional positioning of five cryoprobes and the dissection with air obtained to separate the bowel from the ablation zone. (C, D) CT scans at the end of the procedure depicted perirenal hematoma without active bleeding.

**Figure 7:**
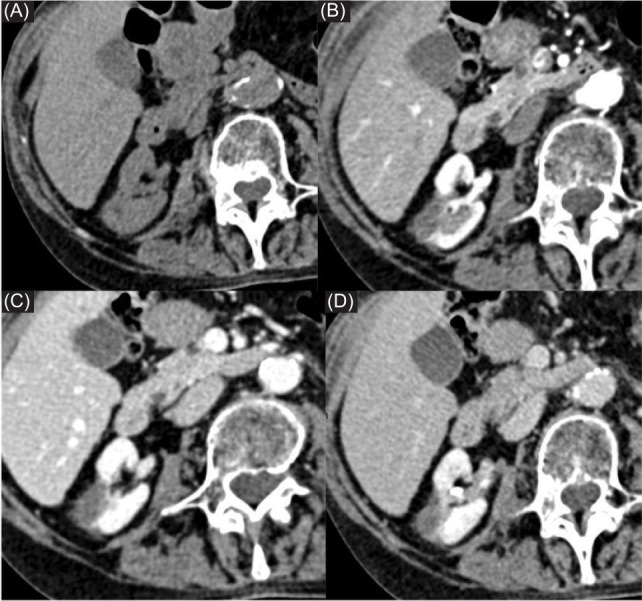
Contrast-enhanced CT scan after 6 months demonstrated complete necrosis area without residual viable tissue.

In March 2020, A 71-year-old female was referred for treatment of an incidental right renal lesion in the presence of macroscopic hematuria. Her history included a lung adenocarcinoma with bone metastases in chemotherapy. Her laboratory data such as complete blood cell count and creatinine demonstrated no significant CHANGES. For appearance of macroscopic hematuria, a contrast-enhanced CT scan was performed revealing a 3.0×2.7-cm oval mass with heterogeneous density in based scan located in the upper pole of the right kidney (Figure 5). It contained sporadic fat with a progressive enhancement observed in a major part of the tumor. Renal biopsy was performed and immunohistochemical findings demonstrated that tumor cells were positive for smooth muscle actin (SMA) and melanocytic markers (human melanoma black 45 [HBM-45] and Melan-A), and negative for desmin, cytokeratin 7 (CK 7), carbonic anhydrase IX (CAIX), and thyroid transcription factor-1 (TTF-1). The renal mass was diagnosed as AML. The patient was qualified for percutaneous cryoablation to reduce her symptoms. The procedure was performed under local anesthesia and deep sedation in the supine position. A total of five cryoprobes (BTG, Boston Scientific) of 17 G were placed into the target area, with assistance of combined CT and augmented infrared navigation system (SIRIO, MASMEC, Italy) (Figure 6) to cover the complete lesion and to spare as much normal parenchyma as possible. The control scan at the end of the procedure demonstrated a perirenal hematoma without active bleeding. The patient was discharged after 2 days of the procedure. All symptoms disappeared after 1 month of cryoablation; the CT control after 6 months demonstrated complete necrosis area without residual viable tissue (Figure 7). In these 6 months, the values of renal function remained within the limits.

## Discussion

For the management of AML, active surveillance remains the first line of option in properly selected asymptomatic patients with small AMLs. However, some of them may cause symptoms like pain or hematuria and may require treatment. The frequency of symptoms increases with the size of the mass. Even if the mass grows, its size increases very slowly and bleeding is a rare event (2.2%) (14). The most frequently reported active treatment is surgery (31%), particularly nephron-sparing surgery (NSS), followed by TAE (17%) (14). Ablative therapies are now utilized more to support renal function preservation and tumor control in short-term follow-up. Unfortunately, TAE is not free from complications. Although complexities connected with the injury of big vessels and renal failure are rare, but others such as post-embolization syndrome, meaning pain and elevated temperature, occur in most patients (22). In the study conducted by Margulis et al. (23), the success rate was 70%, which, in the author’s opinion, can be increased by repeating the procedure. Kuusk et al. established the 3-year rate of freedom from reintervention after embolization to be 63.5% and concluded that among treatment modalities, TAE was associated with a significant higher risk of reintervention (16). Makki et al. established in their study that cryoablation requires no reintervention, with a minor complication rate of only 5.5% and a mean renal AML size reduction rate of 75.6% (21). When comparing current methods for treating renal AML, cryoablation appears to be an attractive alternative to TAE or partial nephrectomy. We decided to employ TAE in the treatment of symptomatic AML measuring >4 cm, and CRA percutaneously in the treatment of small symptomatic AMLs. In such cases, dimension of the lesion is crucial for the choice of treatment, particularly controlling active bleeding in AML requires embolization. Although ablative options make the treatment of subclinical AML compelling, the goals of the treatment are to be defined in a better manner. Both the above-discussed techniques continue to evolve, and since the preservation of renal function remains a priority; modality selection must consider the clinical context and the overall goals for individual patient.

## CONCLUSION

For the treatment of symptomatic AML, cryoablation appears to be a valid alternative to TAE or partial nephrectomy. The choice of treatment must consider the preservation of renal function and the clinical context.

## References

[1] Soulen MC, Faykus MH Jr, Shlansky-Goldberg RD, Wein AJ, Cope C. Elective embolization for prevention of hemorrhage from renal angiomyolipomas. J Vasc Interv Radiol.1994;5(4):587–91. 10.1016/S1051-0443(94)71558-X 7949715

[2] Koo KC, Kim WT, Ham WS, Lee JS, Ju HJ, Choi YD. Trends of presentation and clinical outcome of treated renal angiomyolipoma. Yonsei Med J. 2010;51(5):728–34. 10.3349/ymj.2010.51.5.728 20635448 PMC2908871

[3] Martignoni G, Amin MB. Angiomyolipoma. World Health Organization classification of tumours. In: Eble JN, Sauter G, Epstein JI, Sesterhenn IA, editors. Pathology and genetics of tumours of the urinary system and male genital organs. Lyon, France: IARC Press; 2004. p. 65–7.

[4] Nelson CP, Sanda MG. Contemporary diagnosis and management of renal angiomyolipoma. J Urol. 2002;168(4, Pt 1):1315–25. 10.1016/S0022-5347(05)64440-0 12352384

[5] Oesterling JE, Fishman EK, Goldman SM, Marshall FF. Themanagement of renal angiomyolipoma. J Urol. 1986;135:1121–4. 10.1016/S0022-5347(17)46013-7 3520013

[6] Bissler JJ, Kingswood JC. Renal angiomyolipomata. Kidney Int. 2004;66(3):924–34. 10.1111/j.1523-1755.2004.00838.x 15327383

[7] Lemaitre L, Claudon M, Dubrulle F, Mazeman E. Imaging of angiomyolipomas. Semin Ultrasound CT MR. 1997;18(2):100–14. 10.1016/S0887-2171(97)90054-8 9163829

[8] Sivalingam S, Nakada SY. Contemporary minimally invasive treatment options for renal angiomyolipomas. Curr Urol Rep. 2013;14:147–53. 10.1007/s11934-013-0311-3 23378161

[9] Ramon J, Rimon U, Garniek A, Golan G, Bensaid P, Kitrey ND, et al. Renal angiomyolipoma: Long-term results following selective arterial embolization. Eur Urol. 2009;55:1155–61. 10.1016/j.eururo.2008.04.025 18440125

[10] Hocquelet A, Cornelis F, Le Bras Y, Meyer M, Tricaud E, Lasserre AS, et al. Long-term results of preventive embolization of renal angiomyolipomas: Evaluation of predictive factors of volume decrease. Eur Radiol. 2014;24:1785–93. 10.1007/s00330-014-3244-4 24889998

[11] Murray TE, Doyle F, Lee M. Transarterial embolization of angiomyolipoma: A systematic review. J Urol. 2015;194:635–9. 10.1016/j.juro.2015.04.081 25916674

[12] Staehler M, Sauter M, Helck A, Linsenmaier U, Weber L, Mayer K. Nephron-sparing resection of angiomyolipoma after sirolimus pretreatment in patients with tuberous sclerosis. Int Urol Nephrol. 2012;44:1657–61. 10.1007/s11255-012-0292-z 23054313

[13] Castle SM, Gorbatiy V, Ekwenna O, Young E, Leveillee RJ. Radio-frequency ablation (RFA) therapy for renal angiomyolipoma (AML): An alternative to angio-embolization and nephron-sparing surgery. BJU Int. 2012;109:384–7. 10.1111/j.1464-410X.2011.10376.x 22176671

[14] Fernandez-Pello S, Hora M, Kuusk T, Tahbaz R, Dabestani S, Abu-Ghanem Y, et al. Management of sporadic renal angiomyolipomas: A systematic review of available evidence to guide recommendations from the European Association of Urology Renal Cell Carcinoma Guidelines Panel. Urol Oncol. 2020 2;3(1):57–72. 10.1016/j.euo.2019.04.005 31171501

[15] Muller A, Rouvie`re O. Renal artery embolization: indications, technical approaches and outcomes. Nat Rev Nephrol. 2015;11(5):288–301. 10.1038/nrneph.2014.231 25536394

[16] Kuusk T, Biancari F, Lane B, Tobert C, Campbell S, Rimon U, et al. Treatment of renal angiomyolipoma: Pooled analysis of individual patient data. BMC Urol. 2015;15(1):123. 10.1186/s12894-015-0118-2 26710923 PMC4693425

[17] Kiefer RM, Stavropoulos SW. The role of interventional radiologytechniques in the management of renal angiomyolipomas. Curr Urol Rep. 2017;18(5):36. 10.1007/s11934-017-0687-6 28299630

[18] Castle SM, Gorbatiy V, Ekwenna O, Young E, Leveillee RJ. Radiofrequency ablation (RFA) therapy for renal angiomyolipoma (AML): An alternative to angio-embolization and nephron-sparing surgery. BJU Int. 2012;109:384–7. 10.1111/j.1464-410X.2011.10376.x 22176671

[19] Byrd GF, Lawatsch EJ, Mesrobian HG, Begun F, Langenstroer P. Laparoscopic cryoablation of renal angiomyolipoma. J Urol. 2006;176:1512–6. 10.1016/j.juro.2006.06.013 16952669

[20] Johnson SC, Graham S, D´Agostino H, Elmajian DA, Shingleton WB. Percutanous renal cryoablation of angiomyolipomas in patients with solitary kidneys. Urology. 2009;74:1246–9. 10.1016/j.urology.2008.09.005 19815260

[21] Makki A, Høyerc OGS, Solvigd J, Østraata Ø, Madsena MG, Nielsena TK. Cryoablation of renal angiomyolipoma—An evaluation of safety and efficacy. J Endourol. 2017 11;31(11):1117–22. 10.1089/end.2017.0376 28830229

[22] Matuszewski M, Michajłowski J, Bianek-Bodzak A, Krajka K. Radiofrequency ablation of small symptomatic angiomyolipomas of the kidney: Report of two cases. Pol J Radiol. 2010 7;75(3):68–71. 22802796 PMC3389883

[23] Margulis V, Maatsumoto ED, Lindberg G, Tunc L, Taylor G, Sagalowsky AI, et al. Acute histologic effects of temperature-based radiofrequency ablation on renal tumor pathologic interpretation. Urology. 2004;64(4):660–3. 10.1016/j.urology.2004.05.023 15491694

